# Mammosphere culture of metastatic breast cancer cells enriches for tumorigenic breast cancer cells

**DOI:** 10.1186/bcr2106

**Published:** 2008-06-09

**Authors:** Matthew J Grimshaw, Lucienne Cooper, Konstantinos Papazisis, Julia A Coleman, Hermann R Bohnenkamp, Laura Chiapero-Stanke, Joyce Taylor-Papadimitriou, Joy M Burchell

**Affiliations:** 1 Breast Cancer Biology Group, King's College London School of Medicine, Guy's Hospital Campus, Great Maze Pond, London SE1 9RT, UK; 2 Current address: Centenary Institute of Cancer Medicine and Cell Biology, University of Sydney, NSW 2042, Australia; 3 Current address: Theagenion Cancer Hospital, Al. Symeonidi 2, Thessaloniki 54007, Greece; 4 Current address: MediGene AG, Lochhamer Str. 11, 82152 Planegg/Martinsried, Germany

## Abstract

**Introduction:**

The identification of potential breast cancer stem cells is of importance as the characteristics of stem cells suggest that they are resistant to conventional forms of therapy. Several techniques have been proposed to isolate or enrich for tumorigenic breast cancer stem cells, including (a) culture of cells in non-adherent non-differentiating conditions to form mammospheres and (b) sorting of the cells by their surface phenotype (expression of CD24 and CD44).

**Methods:**

We have cultured metastatic cells found in pleural effusions from breast cancer patients in non-adherent conditions without serum to form mammospheres. Dissociated cells from these mammospheres were used to determine the tumorigenicity of these cultures. Expression of CD24 and CD44 on uncultured cells and mammospheres derived from the pleural effusions was documented.

**Results:**

We found that the majority (20/27) of the pleural effusions tested contained cells capable of forming mammospheres of varying sizes that could be passaged. After dissociation and plating with serum onto adherent dishes, the cells can differentiate, as determined by the increased expression of cytokeratins and MUC1. Analysis of surface expression of CD24 and CD44 on uncultured cells from 21 of the samples showed that the cells from some samples separated into two populations, but some did not. The proportion of cells that could be considered CD44^+^/CD24^low/- ^was highly variable and did not appear to correlate with the ability to form the larger mammospheres. Of eight pleural effusion mammospheres tested in severe combined immunodeficiency disease (SCID) mice, four were found to induce tumours when only 5,000 or fewer cells were injected, whereas the same number of uncultured cells did not form tumours. The ability to induce tumours appeared to correlate with the ability to produce the larger mammospheres. Uncultured cells from a highly tumorigenic sample (PE14) were uniformly negative for surface expression of both CD24 and CD44.

**Conclusion:**

This paper shows, for the first time, that mammosphere culture of pleural effusions enriches for cells capable of inducing tumours in SCID mice. The data suggest that mammosphere culture of these metastatic cells could provide a highly appropriate model for studying the sensitivity of the tumorigenic 'stem' cells to therapeutic agents and for further characterisation of the tumour-inducing subpopulation of breast cancer cells.

## Introduction

It is now clear that normal adult tissues are maintained by the controlled proliferation of stem cells that give rise to progenitor cells producing differentiated progeny [1]. In the mouse mammary gland, the presence of stem cells has been clearly demonstrated by showing that single cells can repopulate a cleared fat pad [2-5]. These cells, termed mammary repopulating units (or MRUs), are found in a ratio of 1:1,400 mammary epithelial cells. Although a similar approach has not yet identified MRUs in human breast epithelial cells, other approaches indicate that pluripotent stem cells are present in the human mammary ductal tree. Thus, by analogy with studies on neuronal stem cells, Dontu and colleagues [6] developed a culture system in which cells derived from reduction mammoplasties were seeded in non-adherent non-differentiating culture conditions. Cells capable of surviving and proliferating in such conditions formed discrete clusters of cells termed 'mammospheres'. Such spheroids were enriched in progenitor cells capable of differentiating along multiples lineages (that is, luminal, myoepithelial, and alveolar).

The concept that the growth of tumours is also based on the division of a stem cell giving rise to differentiated progeny is also receiving considerable support. The development of a tumour could then be due to an imbalance in the symmetric versus asymmetric division of the stem cell, or to phenotypic changes in progeny, which bestow stem cell properties on these cells that normally have a finite life span. The importance of this concept lies in the possible difference in the phenotype of the stem cell, which could allow them to evade killing by the existing therapies [7]. In breast cancer, the mammosphere culture system has now been used to identify, and enrich for, putative stem cells using breast cancer cell lines [8-10], a few oestrogen receptor-positive primary invasive breast tumours [10], and ductal carcinoma *in situ *[11]. However, mammospheres have not been cultured from distant metastases.

An alternative approach has been to attempt to define the surface phenotype of cells that induce tumours in immune-suppressed mice upon injection of very low cell numbers. Taking this approach, Al-Hajj and colleagues [12] described the isolation of breast cancer cells from pleural effusions, which could form tumours in severe combined immunodeficiency disease (SCID) mice by sorting for potential stem cell markers. As few as 100 cells classified as CD44^+ ^CD24^low/- ^could form tumours. Significantly, Ponti and colleagues [10] found that 95% to 96% of cells in mammospheres cultured from cell lines and primary breast tumours stained negatively for CD24. However, the expression of CD44 and CD24 in uncultured primary cell isolates was not reported.

Here, we have examined the ability of metastatic cells in pleural effusions from late-stage breast cancer patients to form mammospheres and determined the *in vivo *tumorigenicity of some mammospheres that could be passaged *in vitro*. We also examined the surface expression of CD44 and CD24 by uncultured cells, using flow cytometric analysis, to attempt to correlate the presence of a CD44^+^/CD24^low/- ^phenotype with the ability to form mammospheres and with the tumorigenic potential of the mammosphere cells.

Our data indicate that mammosphere culture of metastatic breast cancer cells from pleural effusions (some of which have been frozen for more than 20 years) can enrich for tumorigenic cells. The data also suggest that this form of culture, since it enriches for the putative tumorigenic cell, is a feasible approach for isolating these cells for characterisation.

## Materials and methods

### Isolation of cells from pleural effusions

Cells were pelleted by centrifuging pleural fluid diluted with RPMI medium, and the pellet was washed twice with phosphate-buffered saline (PBS), before suspension in RPMI medium without serum. Cells were then collected from the interface after centrifuging over a Ficoll density gradient. Work with pleural effusions was carried out with Central Office for Research Ethics (COREC) approval number 05/Q0704/63.

### Mammosphere culture

Single-cell suspensions of cell lines or cells isolated from pleural effusions were suspended at a density of 40,000 cells per millilitre in Dulbecco's modified Eagle's medium/F-12 containing 5 mg/mL insulin, 0.5 mg/mL hydrocortisone, 2% B27 (Invitrogen Ltd., Paisley, Scotland), and 20 ng/mL epidermal growth factor and seeded into six-well plates (2.5 mL per plate) or T80 tissue culture flasks (10 mL per flask) coated with 1.2% polyhema. Cultures were fed weekly and passaged every 2 weeks. Mammospheres were measured using Zeiss Axiovision software (Carl Zeiss, Jena, Germany). When passaged, mammospheres were harvested, incubated with trypsin for 3 minutes at 37°C, and dispersed by pipetting with a 23-gauge needle. After checking for single cells, the cells were pelleted and suspended in mammosphere culture medium to 40,000 cells per millilitre before replating in non-adherent plates or flasks.

### Differentiation of mammosphere cells

Disaggregated mammospheres were seeded on glass coverslips in mammosphere medium supplemented with 1% foetal calf serum (FCS). Cells were allowed to adhere and differentiate for 5 days before fixing and staining.

### Immunofluorescent staining

Parental cells and differentiated mammosphere-derived cells grown on glass coverslips or mammospheres in suspension were fixed in 4% paraformaldehyde for 10 minutes, washed in PBS, and then permeabilised in 0.1% triton for 5 minutes. After further washing in PBS, cells were incubated for 1 hour at room temperature with neat primary antibody SWA11 (a kind gift from Peter Altevogt) [13], cytokeratin 14 (CK14) [14], CK19 [15], and HMFG2 [16,17] supernatants. Following a wash in PBS, the cells were incubated for 1 hour at room temperature in secondary antibody Alexa 488-conjugated rabbit anti-mouse (Molecular Probes, now part of Invitrogen Corporation, Carlsbad, CA, USA) diluted 1:500 in PBS. Cells were washed with PBS before coverslips were air-died and mammospheres stained in suspension were spun-down with all supernatant removed. Coverslips and cells were then stained and mounted with 10 mg/mL DAPI (4,6-diamidino-2-phenylindole dihydrochloride) (Sigma-Aldrich, Poole, UK) in aqueous mountant (Dako 2972; DakoCytomation, Glostrup, Denmark) and viewed under a Zeiss fluorescence microscope (Carl Zeiss).

### Flow cytometry

For the detection of CD24^low/- ^CD44^+ ^populations in uncultured pleural effusions, cells were stained in 96-well plates in a volume of 50 μL with 2 μL per well of each monoclonal antibody: CD24.PE (ML5), CD44.FITC (G44-26), and ESA.PcPCy5 (BD Biosciences, San Jose, CA, USA). Isotype-matched labelled controls were also used in the analysis. Cells were labelled on ice for 30 minutes and washed twice before analysis in the cytometer.

For comparison of ML5 and SWA11 [13] antibodies for detection of CD24 on uncultured pleural effusion cells, unconjugated antibodies SWA11 (antibody supernatant, generous gift from Peter Altevogt) and ML5 (BD Biosciences) were used at 1:30 dilution and neat, respectively, and detected by a fluorescein isothiocyanate-conjugated rabbit anti-mouse secondary antibody diluted 1:100 (DakoCytomation). Incubation was carried out for 45 minutes on ice per antibody with two washes after each incubation. PBS supplemented with 0.5% bovine serum albumin was used for antibody dilution, washes, and resuspension of the cells. Cells were analysed on an Epics XL (Beckman Coulter, Fullerton, CA, USA).

### Mice and tumour challenge

Female SCID (CB17/ICR-Prkdc SCID/crl) mice, 7 weeks old, were intraperitonealy injected with 0.2 mg etoposide. Seven days later, cells resuspended in matrigel/RPMI were injected subcutaneously in the flank region. Mice were examined every 2 days for the appearance of a tumour, which was measured with callipers. When tumours reached 1.4 cm^2^, they were taken and placed in 4% paraformaldehyde. All animal work was performed under Home Office guidelines and under project licence number 70/4701.

### Immunoperoxidase staining

Xenograft tumours were fixed in formal saline and processed to paraffin wax, and 3-μm sections were cut using a microtome. After sections were air-dried overnight, they were dewaxed in xylene and dehydrated in alcohol. The sections were stained with the following antibodies: anti-keratin 14 (Biogenics, Napa, CA, USA), anti-keratin 19 (BA17 [15]; DakoCytomation), HMFG1 [16,17], HMFG2 [16,17], and SWA11 [13] using the DakoCytomation REAL™ EnVision™ detection system according to the manufacturer's instructions. When staining with anti-CK14 and anti-CK19, antigen retrieval was performed by pressure-cooking for 2 minutes in citrate buffer (pH 6.0) (DakoCytomation).

## Results

### Mammosphere culture of metastatic breast cancer cells from pleural effusions

**Figure 1:**
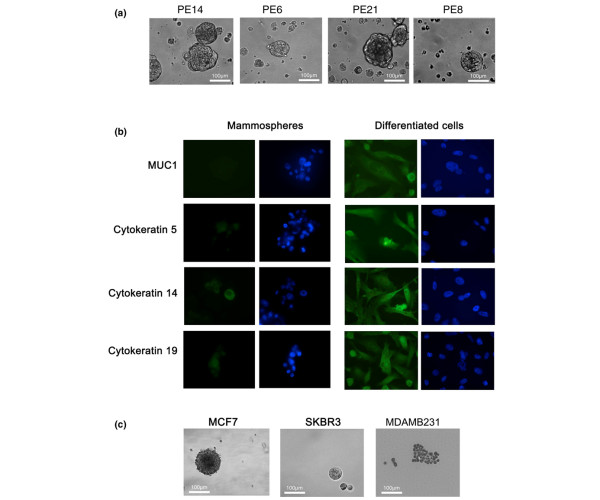
Metastatic cells from pleural effusions isolated from breast cancer patients can form mammospheres. **(a) **Cells were isolated from pleural effusions (PEs) and placed in non-differentiating medium in non-adherent culture flasks (see Materials and methods). PE14, PE6, PE21, and PE8 show four representative cultures. **(b) **PE mammospheres were disrupted and the cells plated onto glass coverslips in medium supplemented with 1% foetal calf serum. After 5 days of adherent culture, the cells were stained with antibodies to MUC1 (HMFG2), CK5 (D5/6), CK14 (LL002), and CK19 (BA17). **(c) **Breast cancer cell lines were also placed in non-differentiating medium in non-adherent culture flasks. Mammospheres could be seen developing in MCF7 and SKBR3 cultures, while MDAMB231 produced loosely adhered clumps of cells.

Single-cell suspensions derived from pleural effusions from 27 breast cancer patients or ascites from 5 breast cancer patients were placed in mammosphere culture. Most of the samples had been frozen for more than 20 years, although five cultures were from fresh specimens of pleural effusions collected in 2006 (designated 06 followed by the PE number in Table 1). Twenty out of 27 pleural effusion samples (74%) produced viable mammospheres (20 to 100 μm) that could be cultured past passage 2 (Table 1). In many cases, these could be passaged further. However, no ascites samples produced viable mammospheres beyond a second passage (0/5). Figure 1a shows mammospheres cultured from four samples, and for comparison, the mammospheres produced by three breast cancer cell lines are shown in Figure 1c. Only the two epithelial cell lines produced discrete relatively large mammospheres, which could be passaged. The fibroblastic-like cell line MDA-MB-231 formed small loosely adherent structures, which, however, did survive passaging.

**Table 1:** Surface phenotype and mammosphere culture of cells from pleural effusions

Sample	Average size of mammospheres (μm)	Population of CD44^+^/CD24^low/- ^cells (percentage)
PE33	No mammospheres^a^	14.2
PE61	No mammospheres	
PE70	No mammospheres	37
PE75	No mammospheres	62
PE77	No mammospheres	18.8
PE78	No mammospheres	10.4
PE89	No mammospheres	
		
PE19	20	67.9
PE20	20	0.3
PE22	20	
PE23	20	65
PE62^b^	20	13.2
PE63	20	
PE88^b^	20	37
PE6^b^	30	1.5
PE79	40	4.8
PE11	50	26
PE43^b^	50	9.9
06PE5^c^	50	
06PE7^c^	50	
		
PE73	60	29.4
06PE4^c^	60	4.9
06PE8^b,c^	70	70.6
PE14^b^	100	Uncultured cells uniformly CD44^- ^and CD24^-^
PE21	100	3.3
06PE6^b,c^	100	15.3
PE66^b^	130	6.85

Cells from pleural effusion (PE) samples taken from breast cancer patients were tested for their ability to form mammospheres. Uncultured cells were analysed for surface expression of CD24 and CD44 (Figure 3). ^a^7/27 (26%) did not make mammospheres or could not be passaged beyond passage 2. ^b^Tested for tumorigenicity in severe combined immunodeficiency disease mice (Table 2). ^c^Five samples obtained in 2006 were placed into culture without freezing. The other 22 samples obtained from 1977 to 1984 had been stored in liquid nitrogen.

To determine whether mammosphere-cultured cells from pleural effusions taken from late-stage breast cancer patients could differentiate into multiple lineages, cells were placed in differentiating conditions and then stained for lineage markers. In the normal breast, different keratins predominate in the different lineages, with CK18 and CK19 being expressed in the luminal cells and CK5 and CK14 in basal/myoepithelial cells. Mammospheres were disrupted, and single cells were plated on glass coverslips in medium supplemented with 1% FCS and after 5 days were stained with antibodies to keratins 5, 14, and 19 and to the luminal cell marker MUC1. While cells from uncultured pleural effusion samples were not viable under these culture conditions, pleural effusion cells that had been cultured first as mammospheres survived. Figure 1b shows that differentiated PE14 mammosphere-derived cells showed higher expression of CK5, CK14, and CK19 compared with undifferentiated mammospheres (Figure 1b). The MUC1 membrane mucin expressed by luminal epithelial cells and by the bulk population of most breast tumours was also not expressed in mammospheres, but expression was induced after transferring the disrupted mammospheres to adherent plates in the presence of serum (Figure 1b).

### CD44 and CD24 expression by uncultured cells and growth of mammospheres from cells in pleural effusions from breast cancer patients

**Figure 2:**
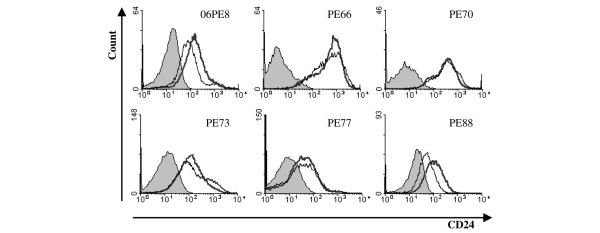
CD24 expression by pleural effusion cells as detected by antibodies SWA11 and ML5. Cells isolated from pleural effusions were stained with unconjugated SWA11 and ML5, and antibody binding was detected by fluorescein isothiocyanate-conjugated rabbit anti-mouse secondary antibody (see Materials and methods). Filled histograms, 2° antibody only; thin grey line, SWA11; thick black line, ML5. PE, pleural effusion.

A CD44^+ ^CD24^low/- ^population was isolated from breast tumours and pleural effusions by Al-Hajj and colleagues [12] and these **cells **were found to be enriched with tumorigenic cells. In these experiments, cells were sometimes gated for epithelial specific antigen-positive (ESA^+^) cells, and there was a suggestion that the ESA^+ ^subset of CD44^+^/CD24^low/- ^cells were more tumorigenic. Using the same CD24 antibody used by Al Hajj and colleagues [12] (ML5) and the same CD44 antibody, we found that only some pleural effusion samples yielded subfractions of cells and these could be detected whether or not the cells were first gated as ESA^+ ^(data not shown). We therefore proceeded to analyse the samples without gating for ESA. We also checked whether there was any difference in staining for CD24 using the SWA11 antibody, which is reactive with all glycoforms of CD24, and ML5, the antibody used by Ponti and colleagues [10] and Al-Hajj and colleagues [12]. Figure 2 shows that, for six pleural effusion samples, we found that SWA11 did not give a stronger signal or detect more CD24^+ ^cells. All the samples were therefore analysed using the phycoerythrin-conjugated ML5 antibody (see Materials and methods).

**Figure 3:**
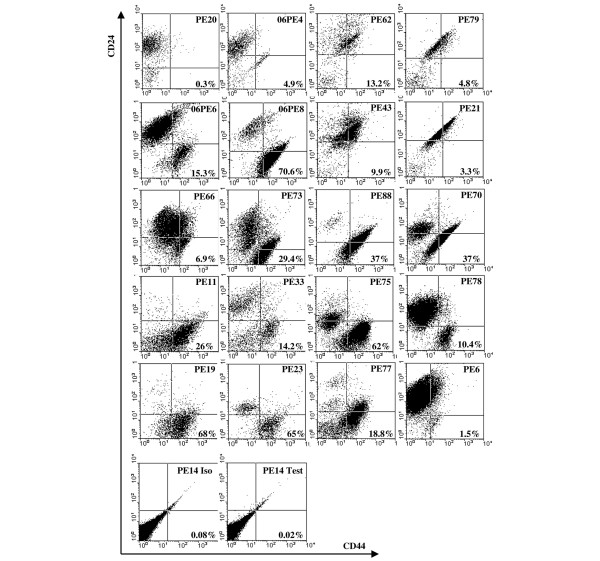
CD24 and CD44 expression by pleural effusion cells. Cells isolated from pleural effusions were analysed for their expression of CD44 and CD24 by flow cytometry using antibody clone G44-26 conjugated to fluorescein isothiocyanate to detect CD44 and using the ML5 antibody conjugated to phycoerythrin to detect CD24 (see Materials and methods). Control analyses were performed with an isotype-matched antibody. This is shown for the sample PE14 (Iso) along with the analysis with CD44 and CD24 antibodies (Test). For the other samples, the lines to form the quadrants were applied based on the analysis with the control antibody. The percentages in the right bottom quadrant refer to the CD44^+ ^CD24^low/- ^population. PE, pleural effusion.

Figure 3 records the fluorescence-activated cell sorting profiles of uncultured cells stained with antibodies to CD24 and CD44 for 21 of the 27 samples of pleural effusions. The data show that in several samples two distinct populations can be recognised (for example, PE06/8, PE88, and PE70), whereas with other samples this is not the case. Moreover, CD44^+^/CD24^low/- ^cells were often within a subpopulation and existed only as a discrete defined subset in some samples (for example, PE23 and PE75). Inserting the cutoff values from the control antibody analysis, the cells in the bottom righthand quadrant were considered as CD44^+^/CD24^low/-^, and the percentage for each sample is listed in Table 1 and Figure 3. Comparing the ability to form mammospheres and the size of the mammospheres produced with the presence of a CD44^+^/CD24^low/- ^population as defined in this way showed no obvious correlation with the ability of the cells to form mammospheres or with the size of the mammosphere produced (Table 1).

**Figure 4:**
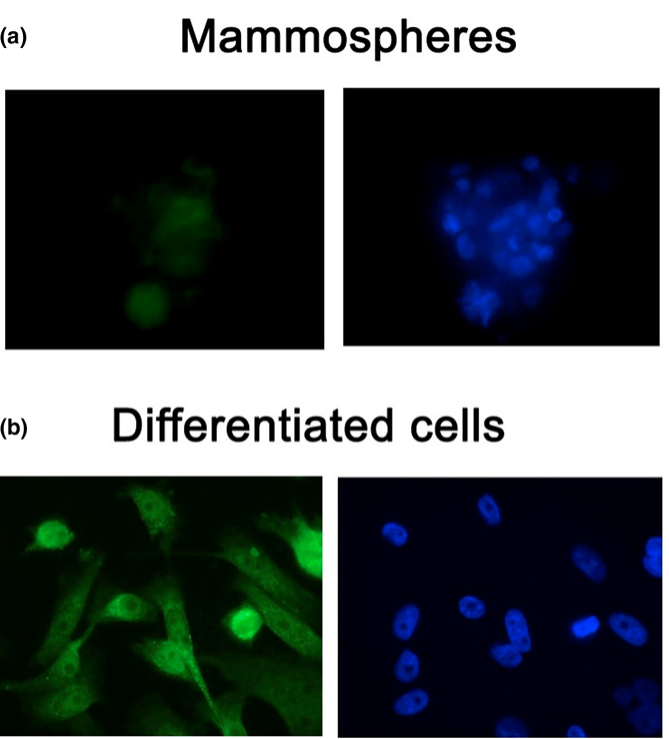
CD24 expression in mammospheres. **(a) **Mammospheres from pleural effusion PE14 and **(b) **cells from disrupted PE14 mammospheres differentiated in the presence of serum were stained for CD24 expression using the SWA11 antibody, and binding was visualised using rabbit anti-mouse Alexa 488-conjugated antibody (left panels). Right panels are the same cells stained with DAPI (4,6-diamidino-2-phenylindole dihydrochloride). PE, pleural effusion.

The flow profile of the PE14 sample was particularly striking as surface expression of CD24 and CD44 by the uncultured cells was uniformly negative (Figure 3). Moreover, mammospheres from this sample were large and could be extensively passaged. Staining of PE14 mammospheres for CD24 with either the SWA11 or ML5 antibody showed that CD24 was low or absent (Figure 4a), as was also observed by Ponti and colleagues [10] in mammospheres cultured from recurrent breast cancer or MCF7 cells. As with the lineage markers, expression was increased upon differentiation (Figure 4b).

### Tumour growth *in vivo*

**Figure 5:**
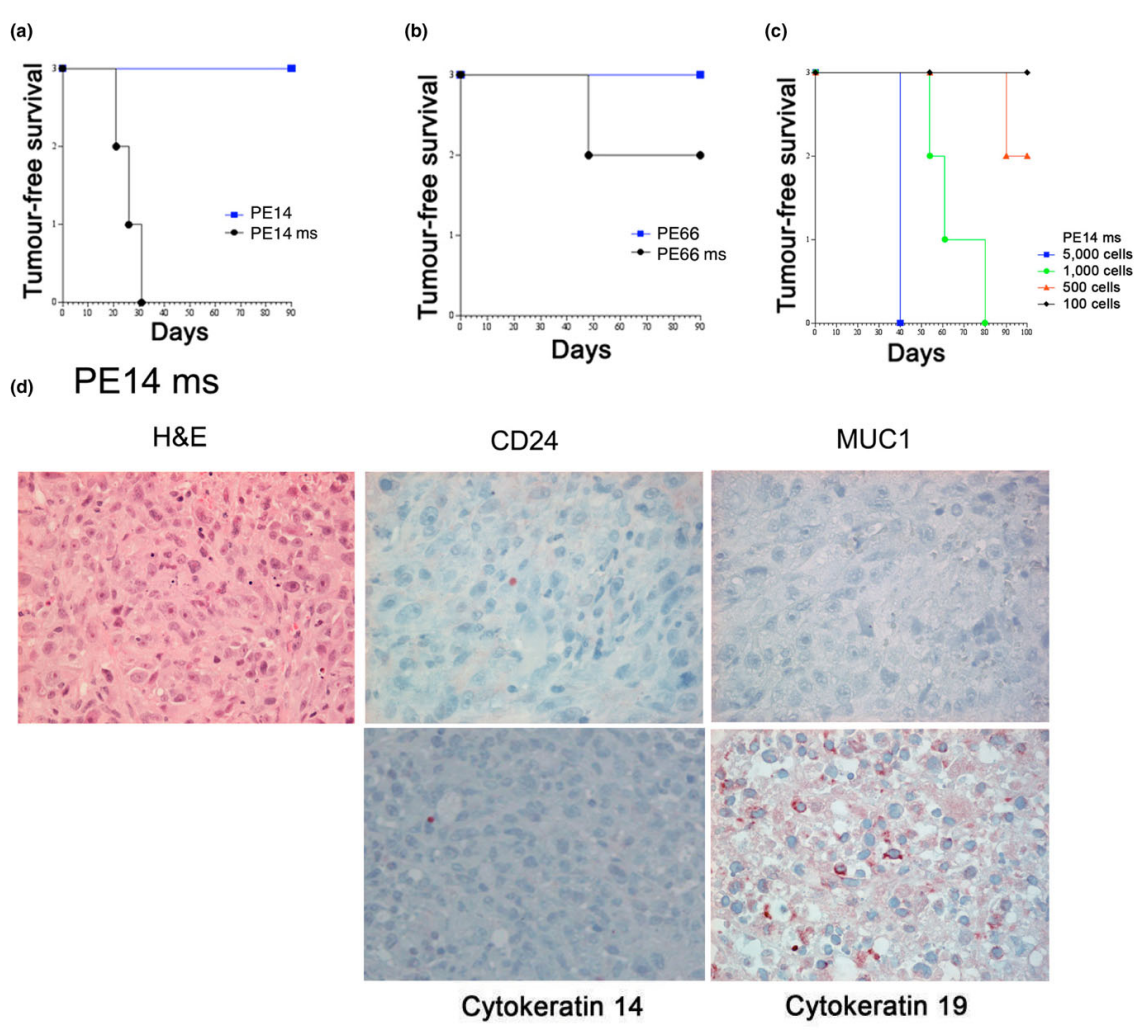
Mammosphere-derived cells from pleural effusions are tumorigenic. Five thousand uncultured or mammosphere-derived cells were injected into severe combined immunodeficiency disease mice, and tumour development was monitored. Survival graphs for PE14 **(a) **and PE66 **(b) **are shown. **(c) **Survival curves of mice injected with different numbers of cells (as indicated) derived from PE14 mammospheres. **(d) **Tumours derived from cells isolated from PE14 mammosphere cultures were removed, fixed, sectioned, and stained for CD24 (SWA11), MUC1, and CK14 and CK19 expression, as described in Materials and methods. CK, cytokeratin; H&E, haematoxylin and eosin; ms, mammosphere; PE, pleural effusion.

To assess tumorigenic potential, cells from mammospheres from selected samples (governed by availability) were injected into SCID mice (Table 2). The mammospheres were gently disrupted by pipetting and resuspended in matrigel solution. Cells were injected into the flank of mice that had been previously injected intraperitonealy with etoposide solution, but the mice did not receive oestrogen implants. Unsorted/uncultured cells were injected as a control. The number of cells available for injection restricted the number of mice and the number of cells that could be injected. To obtain the data shown in Table 2, 5,000 cells from mammospheres or uncultured cells from eight samples were injected per mouse. Whereas four of the samples tested in this way produced tumours, uncultured cells from these samples did not (Figures 5a and 5b and Table 2). Figures 5a and 5b show the tumour development in mice injected with 5,000 cells from mammospheres grown from samples PE14 and PE66. Cells from PE14 mammospheres were extremely tumorigenic and cell dilution experiments showed that tumours could be produced from as few as 500 cells (Figure 5c).

**Table 2:** Tumorigenicity of cells from pleural effusion mammospheres

Sample	Average size of mammospheres (μm)	Percentage of CD44^+^/CD24^low/- ^cells	Tumorigenicity
PE88	20	37	0/3^a^
PE62	20	13.2	0/3
PE6	30	1.5	0/3
PE43	50	9.9	0/3
06PE8	70	70.6	4/7
06PE6	100	15.3	1/3
PE14	100	All cells CD24^- ^and CD44^-^	5/7
PE66	130	6.8	1/3

Mice were injected with 5,000 cells from disrupted mammospheres as described in Materials and methods. ^a^The ratio of mice developing tumours over mice injected. PE, pleural effusion.

Table 2 shows that of the four samples that induced tumours, all produced mammospheres larger than 50 μm; however, tumorigenicity did not correlate with the percentage of CD44^+^/CD24^low/- ^cells in the original population. Uncultured cells from the fourth sample, PE14, showed the unusual feature of expressing no surface CD24 or CD44. Moreover, tumours isolated from the mouse did not express these components either, nor did they express the lineage markers MUC1 or CK14 (Figure 5d) in accordance with the pathology of the tumours, which showed no indication of differentiation but a high percentage of mitoses. Weak keratin 19 expression was detected (Figure 5d), and while this is a luminal marker, it has been reported to be expressed by stem cells [18].

## Discussion

The hypothesis that tumours arise in the stem cells of tissues rather than the differentiated cell lineages is not new [19,20], but it is only recently that the tools have become available to evaluate this hypothesis. [21,22]. Two of the major challenges in these studies, particularly in breast cancer, are (a) to identify breast cancer stem cell markers and (b) to isolate the presumably limited number of cells that can recapitulate a breast tumour *in vivo*. CD24^low/- ^CD44^+ ^has been suggested to define a population of cells that contain potential breast cancer stem cells [12], as has high expression of α-6 integrin [8]. On the other hand, culturing cells in so-called 'mammosphere culture' has been proposed as a method for enriching the progenitor cell pool *in vitro*. With this background, we examined cells in samples of pleural effusions for the presence of a CD44^+^/CD24^low/- ^subpopulation and for their ability to grow in non-adhesive conditions as mammospheres. While cells with a CD44^+^/CD24^low/- ^phenotype could be detected in some samples, the percentage of such cells was highly variable between samples. In some cases, the CD44^+^/CD24^low/- ^phenotype formed a discrete population, but in others the phenotype was absent or was part of a larger subgroup. Moreover, the ability to form the larger mammospheres did not appear to correlate with the percentage of cells with this phenotype but did correlate with the ability of the mammosphere cells to induce tumours in SCID mice. Our data suggest that enrichment of tumorigenic cells from the bulk tumour population may be most readily achieved by mammosphere culture.

It is of note that the majority of the samples examined had been stored in liquid nitrogen for more than 20 years. Although most of these samples were able to generate mammospheres (17/22), the majority were small in size (<50 μm). Of these 17, seven pleural effusion samples gave rise to mammospheres that had an average diameter of only 20 μm, which may be on the borderline of the definition of a mammosphere. Nevertheless, mammospheres larger than 50 μm were generated from five of these stored samples, two of which were shown to induce tumours in SCID mice (PE66 and PE14). Samples collected recently and cultured directly (06 series) all gave mammospheres of a reasonable size (≥ 50 μm) and the two tested induced tumours in mice. Thus, although the stored tumour cells from some samples were highly proliferative and tumorigenic, some produced no mammospheres or were small. The results suggest that in the unfrozen samples collected recently, the survival of the tumour-inducing mammosphere-producing cells may have been more consistent than in the stored samples. If this is so, freezing could have affected the recovery of the CD44^+^/CD24^low/- ^cells in some samples. It may be that conditions for freezing the tumorigenic cells need to be optimised if samples are to be frozen.

One of the stored pleural effusions, PE14, which formed large mammospheres (average 100 μm) that were highly tumorigenic, showed an unusual phenotype in that all of the cells in the uncultured population were negative for surface expression of CD24 and CD44. The fact that all the cells were negative for CD24 could indicate that the stem cell was highly imbalanced toward symmetrical cell division. The absence of the CD44 surface marker was unexpected but could reflect the true phenotype of the cancer stem cell, the CD24^low/- ^CD44^+ ^profile reflecting the phenotype of progenitor cells. The tumours developing in the mouse injected with PE14 mammosphere cells also showed no expression of CD24 or of most lineage markers, being in agreement with the pathology, which showed an undifferentiated highly mitotic tumour. Weak expression of keratin 19 was detected in the PE14 mouse tumours, but this has also been reported to be a marker for the cancer stem cell [18]. The fact that tumours were not induced by low numbers of uncultured PE14 cells, however, indicates that selection for the tumorigenic cells has occurred in the mammosphere culture. In contrast to the phenotype of the tumours in the mice, the pluripotency of the PE14 mammosphere cells could be demonstrated by culturing them under differentiating conditions, when the class lineage markers were expressed.

## Conclusion

This report demonstrates the presence in metastatic pleural effusions from breast cancer patients of cells capable of forming mammospheres that after dissociation can differentiate, expressing markers of both luminal and basal cells. Some of these mammospheres have the ability to form tumours in SCID mice after the injection of very small numbers of cells (in one case, as low as 500), which appears to correlate with the size of the mammosphere. Xenograft models of human breast cancer have traditionally been very hard to establish and maintain. The high success rate for culturing accessible metastatic breast cancer cells as mammospheres suggests that mammosphere culture could provide a highly appropriate model for studying the sensitivity of the tumorigenic 'stem' cells in the tumour to therapeutic agents. A comparison of efficacy using mammospheres from breast cancer cell lines or mammospheres cultured from individual pleural effusions as targets would allow evaluation of the consistency of effectiveness of a specific therapy.

## Abbreviations

CK = cytokeratin; ESA = epithelial specific antigen; FCS = foetal calf serum; MRU = mammary repopulating unit; PBS = phosphate-buffered saline; PE = pleural effusion; SCID = severe combined immunodeficiency disease.

## Competing interests

The authors declare that they have no competing interests.

## Authors' contributions

MJG performed some of the experiments and contributed to the first draft of the paper. LC cultured the mammospheres and performed the immunohistochemistry. KP performed the first flow cytometric analysis of the pleural effusion. JAC performed flow cytometric analysis of the pleural effusions. HRB isolated cells from pleural effusions and assisted with flow cytometric analysis. LC-S assisted with isolating cells from pleural effusion and culturing of the mammospheres. JT-P wrote the first drafts of the original and resubmitted manuscripts and supervised the work. JMB supervised the work and contributed to the final drafts of the manuscripts. JT-P and JMB contributed equally to this work. All authors read and approved the final manuscript.
